# Dietary Behavior and Diet Interventions among Structural Firefighters: A Narrative Review

**DOI:** 10.3390/nu14214662

**Published:** 2022-11-04

**Authors:** Margaux J. Joe, Irene E. Hatsu, Ally Tefft, Sarah Mok, Olorunfemi Adetona

**Affiliations:** 1 Department of Environmental Health Sciences, College of Public Health, The Ohio State University, Columbus, OH 43210, USA; 2 Department of Human Sciences, The Ohio State University, Columbus, OH 43210, USA; 3 OSU Extension, The Ohio State University, Columbus, OH 43210, USA

**Keywords:** firefighter, diet, dietary intake, food, nutrients, calories

## Abstract

Structural firefighters (SFFs) are exposed to multiple occupational hazards that affect dietary behavior and can contribute to increased risks of cancer and cardiovascular disease compared to the United States’ general population. Dietary behavior is a feasible modification for positive health outcomes. The objectives of this narrative review are to summarize the diet behavior of SFFs, review findings of diet interventions that positively modify diet behavior, identify research gaps, and suggest recommendations for addressing those gaps. PubMed, MEDLINE, Scopus, and CABI Web of Science were searched between February 2020 and June 2022 for peer-reviewed articles. The inclusion criteria were as follows: (1) study population must include SFFs; (2) investigate diet or diet intervention among SFFs; (3) report results specific to SFFs; and (4) be published in the English language. Thirty-four studies were included. Results indicate that SFFs recognize the importance of a healthy dietary pattern, but do not follow one, and that food choices are often influenced by colleagues. Diet interventions, such as the Mediterranean diet, were observed to have positive health improvements, such as improved lipid levels and lower CVD risk. Team counseling was found to be more effective for adopting healthier diets compared to one-on-one counseling; and general counseling was more effective than no counseling. A gap identified by this review is the lack of information concerning differences in dietary intake, diet quality, and dietary behaviors while on- and off-shift, and throughout the career. Diet is an important risk factor for occupational disease development; therefore, effective, consistent dietary interventions are necessary.

## 1. Introduction

The U.S. Fire Administration estimates that 96% of registered fire departments are staffed by both career SFFs and volunteer SFFs, and the remaining 4% are staffed by state and federal governments, contractors, private entities, industrial fire brigades, transportation authorities, and airport fire departments [1]. As defined by the Federal Emergency Management Agency (FEMA), a career SFF is an individual “that provides structural fire suppression” [2]. In contrast, a volunteer SFF is an individual who volunteers, or is on-call, instead of being employed by a fire department full-time [3]. As of July 2022, there was a total of 1,062,900 active career and volunteer SFFs in the U.S., with 34% being career SFFs, 54% unpaid volunteer SFFs, and 12% paid-per-call volunteer SFFs; of the registered fire departments 9.2% are fully career SFFs, 4.8% are majority career SFFs, 15.7% are majority volunteer SFFs, and 70.2% are fully volunteer SFFs [1].

SFFs are responsible for fire suppression in buildings, residences, and other facilities [2]. Firefighting operations include a variety of tasks such as running water supply from hydrants, forming attack lines to extinguish flames, executing forcible entries, carrying equipment up multiple flights of stairs, rescuing and evacuating occupants, and extracting victims from vehicles [4]. Due to their responsibilities, SFFs are exposed to a multitude of occupational stressors that can affect their health such as extreme heat, exhaustion, combustion byproducts (e.g., heated gases, vapors, particulate matter), and physical and emotional stress [5]. Other work-related factors influencing the health outcomes of SFFs include shift work, sleep disruptions, irregular mealtimes, and consumption of highly processed foods with excess amounts of sodium and sugar [6,7].

As a result of these occupational exposures and stressors, SFFs have a higher risk of adverse health outcomes such as cancer, hearing loss, rhabdomyolysis, and cardiovascular disease (CVD) [8]. CVD and cancer are the major adverse health outcomes affecting firefighters. Compared to the general U.S. population, SFFs have a 9% increase in being diagnosed with cancer and a 14% increase in cancer death [9,10]. Research has shown that CVD risk factors are more prevalent in SFFs compared to the general population, and SFFs often have excess strain on their cardiovascular system from their working environment and equipment, putting them at an increased risk of a cardiac event [11,12,13]. CVD is the leading cause of on-duty SFF death and accounted for 44% of firefighter fatality in 1995 [14,15]. In 2019, there were 34 fatalities among career SFFs and 25 among volunteer SFFs [16]. Thirty-three of those fatalities were the result of a heart attack. 

Dietary behavior is a major risk factor for development of occupational diseases and is one that is highly modifiable. Multiple studies have observed the dietary behavior of SFFs and have evaluated various dietary interventions to understand if and what methods work best for improving dietary behavior among SFFs. However, the results of these studies are not conclusive. Therefore, to understand the dietary behavior and effective diet interventions among SFFs, the existing peer-reviewed literature was synthesized. The objectives of this review are to summarize the diet behavior of SFFs, review the findings of diet interventions that positively modify diet behavior, identify research gaps, and suggest recommendations for addressing those gaps.

## 2. Materials and Methods

### 2.1. Study Elegibility Criteria

Studies that met the following criteria were included in this review: (1) study population must include SFFs; (2) study must investigate diet or diet intervention among SFFs; (3) study must have reported results specific to SFFs; and (4) study must be published in the English language.

### 2.2. Search and Selection Process

Scientific research databases were electronically searched between February 2020 and June 2022 for eligible peer-reviewed articles using either an observational or intervention study design. These databases include PubMed, MEDLINE, Scopus, and CABI Web of Science. “Firefighter” in combination with each of the following words or phrases were used as keywords for the literature search: “diet”, “dietary intake”, “food”, “food intake”, “nutrients”, “intake”, and “calories”. Boolean operators were not used in the search process. Abstracts were screened for selection using the study criteria by two reviewers. If there was uncertainty that a paper qualified based on reading the abstract, the whole text was reviewed by the reviewers for selection.

Data were initially abstracted from each selected paper by two reviewers and then constructed into a standardized form. The abstracted data were then reviewed by two other reviewers for completeness and accuracy. Data extracted from the selected observational papers include study design, study objective, sample size, source population, age, career length, sex, diet pattern assessment tool, study results, evidence of bias, diet practice context, representativeness, and objective verification. Data extracted from the selected intervention papers include study design, study objective, sample size, age, career length, sex, type and length of intervention, intervention outcome(s), study results, evidence of bias, and food record method, adherence to intervention. Evidence of bias was extracted for all studies using a qualitative narrative approach to assess the risk of bias.

## 3. Results and Discussion

**Figure 1:**
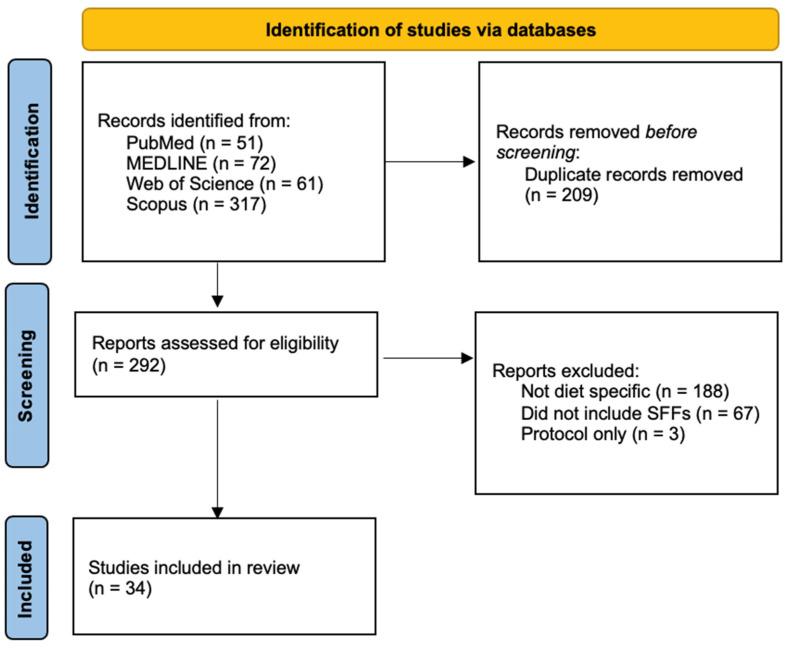
Study selection.

Thirty-four papers met the inclusion criteria for this review (Figure 1). Of these, seventeen were observational studies and seventeen were intervention studies. Characteristics by study type are presented in Table 1 and Table 2. Study limitations and potential biases associated with the study designs of the included studies are also presented in Table 1 and Table 2. Multiple studies had potential risk of information bias due to self-reported data, recall, and possible differential desire to answer questions accurately. Convenient sampling, small sample size, and non-independent samples contributed to selection bias across several of the included studies. Differential, non-differential, and exposure misclassification also contributed to potential risk of bias and measurement error across these studies.

### 3.1. Observational Studies

Seventeen studies of SFF dietary observations are included in this review. Fourteen are cross-sectional in design, one utilizes a fixed mixed methods design, one uses a case-control design, and one is cross-over in design. Twelve of the studies did not have objective verification of SFF dietary habits. Information bias is likely across all studies due to the nature of their design, sample size, lack of representation for both volunteer and career firefighters that affects generalizability, or missing context around the food culture of the SFF work environment.

Of the seventeen observational studies included in this review, ten studies utilized food frequency questionnaires (FFQ) to evaluate diet and lifestyle practices, two utilized food diaries, two studies used both food diaries and FFQs, and three studies conducted in-person interviews that collected information about food perceptions, barriers, and solutions to diet habits. Evidence from the literature indicates that SFFs have a high consumption of red meat and fast food contributing to the insufficiency of consumed calories relative to the energy expenditure needed for firefighting [6,23]. SFFs, during their shift, have been observed to over consume fat, cholesterol, protein, sugar, and sodium, and under consume fruits, vegetables, whole grains, and dietary fiber than is recommended [24,31]. More recent studies also indicated that SFFs consume inadequate amounts of linolenic fatty acids, fiber, potassium, magnesium, zinc, and vitamins D, E, and K with vitamin D, magnesium, and potassium having the greatest shortcomings [26,27]. In some environments, the SFF who cooks for the crew determines what the meal is regardless of its quality [21]. SFF job constraints such as having to eat quickly, night calls, and sleep disruptions can also play a role in nutrition and the quality of health [18,21]. Moreover, they are exposed to occupational factors, such as shift work that is 10, 14, 24, or 48 h long [48], which are not easily changed given the nature of the job. Focus groups conducted with SFFs have indicated that shift work, specifically night shifts, often leads to unhealthy dietary behaviors due to a lack of availability of healthy foods at night [29]. These constraints may consequently lead to a higher risk for CVD and cancer [4]. Other occupational factors found to affect the dietary behaviors of SFFs include heat exposure and amount of sleep. Gupta et al. [30] found that SFFs who lacked sleep in addition to being exposed to extreme heat consumed more calories compared to the group who only lacked sleep and the control group of SFFs who were not exposed to any of the two risk factors. It is consistent across studies that a workplace approach to dietary intake is very important because SFFs eat unhealthier meals at work than at their own homes, highlighting the need for direct nutritional guidance and intervention [18]. Barriers to improving nutrition and diet among SFFs include the lack of financial and other resources, nutrition/diet expertise, and a positive community of senior workers [18].

Research has suggested that greater adherence to the Mediterranean diet has substantial inverse correlation with risks for heart disease and diabetes, LDL cholesterol, and weight gain, and is associated with higher HDL cholesterol [20,25,49]. A recent study found that high intakes of anthocyanins (polyphenols found in red, blue, and purple foods) increase HDL cholesterol, suggesting that it may have cardioprotective effects [28]. While this study lost statistical significance in its multivariable models, it does provide significant implications for future studies. Based on their results, Hershey et al. [28] also indicate that the Mediterranean diet, which is rich in polyphenols, can increase anthocyanin intake. 

The potential for heavy alcohol consumption among SFFs is also of concern since excessive use can lead to the development of CVD, liver disease, digestive issues, and cancer [50]. Heavy episodic drinking is prevalent among younger SFFs, with a greater tendency to follow these patterns on their selected day off [19]. It is suggested that alcohol is used to promote social interaction and camaraderie, as well as to cope with work stress or repeated trauma exposure. 

Although the importance of healthy eating and the need for nutritional programs is often acknowledged by SFFs, the majority do not follow a healthy dietary pattern [22]. Those that typically follow a healthy diet pattern tend to be in the normal BMI range. It should be noted that age and BMI are correlated, with older SFFs likely to be in the higher BMI range [17]. Differences in area of service, workload, and work intensity can also contribute to this correlation [22]. Interviews with SFFs have indicated that health promotion beginning at the fire academy can potentially lead to sustained healthier diet patterns in the long-term future [18]. In this study, the authors found that there is a need for fire academies to develop a curriculum that facilitates long-term change, and that promotes incentives for making good dietary choices and healthier decision-making [18]. While observational and intervention research of dietary behavior at fire academies is limited, this is an important finding for understanding the long-term sustainability of positive dietary behaviors.

### 3.2. Intervention Studies

As observed in the selected studies of SFFs, diet intervention protocols vary and may involve educational materials, counseling, dietary programs that are tailored to fit an individual’s needs and lifestyle, or some combination of these. Diet interventions differ in length and for the purpose of this review, study lengths were defined as short term (less than one month; two studies), medium term (one to six months; four studies), or long term (greater than six months; eleven studies). The outcomes of the intervention studies included measures of healthier eating habits and improved physical activity, and/or improved physical characteristics.

All the 17 studies of dietary intervention among SFFs that were identified are randomized trials or cohort studies in design. Most of the intervention studies were conducted in North America, with the exception of one study conducted in Brazil, one study conducted in Hong Kong, and one study conducted in Switzerland. All studies were either composed of majority male participants or did not report demographic information, and none reported gender differences. Seven studies controlled for potential confounders and implemented inclusion and exclusion criteria for selection into the study. Only three studies required participants to maintain food diaries to record dietary intake. Ten studies analyzed adherence to the intervention via biomarker measurements, anthropometric measurements, and/or calculation of the Mediterranean Diet score. No study addressed differences in behavior or the effect of intervention on diet between the fire station and off-work days. Sustainability of diet intervention among SFFs was investigated in only one study.

“Promoting Healthy Lifestyles: Alternative Models’ Effects” (PHLAME) is an intervention designed to encourage healthier eating and exercise behaviors among SFFs given their inherent risk for adverse health outcomes due to occupational exposures [33]. PHLAME was administered in two formats: (1) a team-based curriculum and; (2) one-on-one motivational interviewing (MI) by a trained health counselor. The team-based curriculum focused on SFFs participating in activities involving nutrition, exercise, and other topics that were of interest to the group. The one-on-one MI used a more individualized approach based on the needs of the SFF. The intervention was implemented across four separate randomized control trials in Elliot et al. [34], MacKinnon et al. [40], Pirlott et al. [38], and Ranby et al. [33]. The PHLAME study assessed both short- and long-term outcomes to determine the effectiveness of the interventions. The short-term outcomes included increased fruit and vegetable consumption and decreased weight gain [33,34,38], while the long-term outcomes included improvements in mood, productivity, and perceived well-being [34], as well as sustained improvements in general nutritional and physical behaviors several years following the program [40]. In general, changes due to the intervention were consistent across the four studies: the intervention increased knowledge about healthy diets and the consumption of fruits and vegetables [33,34,38,40]. 

The original PHLAME participants were followed annually for four years, and the primary objective of MacKinnon et al. [40] was to examine the long-term effect of PHLAME after four years of follow-up. At one year of follow-up, team-based participants, compared to MI participants and control participants, showed the most promising upward trajectory of increased fruit and vegetable consumption, greater dietary support, and increased knowledge of healthy dietary habits. However, it was observed that these differences between the team-based participants and the MI and control groups dissipated at later follow-ups; this is most likely due to diffusion of the intervention to the MI participants and control groups throughout the follow-up years since adopting uniform behavior is a common tradition among SFFs [40]. In addition, all study groups were recruited from the same five fire departments in northern Oregon and southern Washington [40]. While the intervention benefit seems not to have been sustained, this finding is important because positive results of diet interventions are consistently reported during and immediately following the completion of the intervention program. However, very few of the studies have examined whether self-efficacy of changed behavior is persistent following the intervention, as was observed for SFFs in this review.

In general, individual health assessments and the provision of feedback on dietary patterns, fitness levels, and body weight increase awareness of potential negative dietary habits but have a very limited effect on dietary behavior change [40]. However, having an intervention in a team setting compared to individual counseling encourages more positive change in behavior [33,34,40]. The four PHLAME-based studies reported that all intervention groups experienced immediate dietary benefits, including improved dietary understanding and increased vegetable and fruit intake, with SFFs in a team setting showing greater widespread effects. Based on these results, it can be inferred that team culture is an important influence in diet culture among SFFs, especially since there are communal mealtimes, peer bonds, and shared responsibility [51]. Furthermore, Elliot et al. [34] suggests that materials for a team curriculum can be significantly cheaper, effective, exportable, and economically feasible compared to MI due to high costs of personal counselor trainings and time spent. With a positively changed attitude towards more optimal diet practices in a crew, behavioral change can be done through leading by example and can set the standard for future SFF crews.

Pirlott et al. [38] conducted the PHLAME intervention through counselor motivational interviewing (MI) only. The authors reviewed a handful of counseling sessions and coded characteristics of how the counselor interacted with the SFFs between MI-consistent behaviors and MI-inconsistent behaviors. Consistent behaviors were classified as the “combined counts of the following utterance categories: affirm, advise with permission, emphasize control, ask open question, reflect, reframe, and support”. MI-inconsistent behaviors were classified as “confront, advise without permission, direct, raise concern without permission, and warn”. Their results indicated that counselor behavior was a predictor of SFF’s intentions to make positive changes such as increased fruit and vegetable consumption. The nuances of how a counselor speaks to the SFFs showed differences; counselors who worked with MI-consistent behaviors (i.e., counselors who utilized positive speech and interaction cues) had a more positive correlation to positive change talks and self-exploration regarding dietary behavior. These results also indicate general improvement among SFFs, regardless of whether it was a counselor or SFF colleague/superior giving encouragement for healthier eating.

Diets involving carbohydrate restriction have also been studied among SFFs. Waldman et al. [32] performed a 28-day carbohydrate-restricted diet (CRD) intervention and examined its effects on SFF performance and their cardiometabolic markers. The performance outcomes measured included a graded cycling test, Wingate test, and a fitness assessment. Results showed up to a 2.7 kg reduction in weight among the firefighters, as well as reductions in fat mass, respiratory exchange rate, systolic blood pressure (SBP) and diastolic blood pressure (DBP), rating of perceived exertion, and a 2.41 km run time. However, the sustainability of the intervention is not clear considering that firefighting requires a lot of expendable energy and carbohydrates are good energy sources. While this intervention mimics the ketogenic diet and promotes weight loss, there are risks if practiced long-term such as hypoproteinemia, fatty liver disease, kidney stones, and vitamin deficiency [52]. Moreover, it may lead to CVD risks due to increases in LDL cholesterol and very-low-density lipoprotein [52]. Another study by Waldman et al. [47] used biomarker measurements to further assess the utilization of a 28-day CRD to improve cardiometabolic health. Their results suggest that a 28-day CRD can improve biomarkers of CVD. However, these improvements were mainly observed in professional male SFFs [47].

Metabolic biomarkers and oxidative stress indicators were investigated by Macedo et al. [36] among Brazilian military firefighters after daily 100 mg resveratrol (RES) intake for three months, which we consider to be a medium-term intervention. RES is a natural plant compound that acts as an antioxidant and is found in natural foods such as grape skins, peanuts, and blueberries. Moderate consumption of red wine, a part of the Mediterranean diet, also contributes to RES in the body, which has been associated with lower CVD incidence [53]. Physical performance assessment was used to induce oxidative stress pre- and post-intervention so that differences in plasma metabolic response and indicators of oxidative stress could be measured before and after the fitness test both pre- and post-intervention. Aside from a decrease in IL-6 and TNF-a in the treatment group from pre- to post-resveratrol intervention, no other significant changes were observed due to the intervention. Furthermore, the authors suggest that the fitness test applied in this study was not sufficient enough to challenge the antioxidant system and therefore the intake of 100 mg of RES for three months may not have been enough to induce effects that is significant enough to demonstrate the impact of resveratrol [53]. In addition, there was no objective compilation to evaluate adherence to supplements. While not addressed in the study, the lack of adherence data may have confounded the findings as no treatment effect on SFF health was observed at the end of the program. Moreover, this study posed a limitation in terms of its real-life application, because SFF at-home medication compliance is low even when there are immediate health consequences, including physician-required medication regimens [54].

General lifestyle and wellness program interventions tailored for SFFs aim to reduce dietary risk factors that are linked to CVD [35]. Such changes include reductions in body weight and body fat, waist circumference, triglyceride and insulin concentrations, systolic and diastolic blood pressure, and resting heart rate; as well as increases in bicep strength and flexibility [35,37]. These interventions may be aided with coaches and/or specialists that maintain one-on-one contact with firefighters through telephone, email, or text communication. Furthermore, seminars and demonstrations have been used to promote health and nutrition education. Goheer et al. [39] had monthly 90-min education sessions that introduced new foods and demonstrated healthier meal preparations while avoiding the stigma of unappealing diet foods. Healthier options for popular restaurants as well as discounted prices on healthier food options were presented to incentivize SFFs’ purchases when eating out. Following the intervention, respondents reported a higher willingness to try new foods or preparation methods, positive changes in the structural SFF food environment, and positive changes in personal home food environment.

Bucher et al. [46] conducted an intervention among firefighters at a Swiss airport that supplemented a baseline prevention program implemented by the airport. They assessed the eating habits of participants and compared the study results to the national guidelines for healthy eating. The overall prevention program that was offered to all airport employees focused on healthy eating and physical activity, and included a one-hour education session, fifteen minutes of individual coaching from a dietician, as well as offers and events geared towards improved eating habits. Because of the specific team organization of airport firefighters and their unique challenges, firefighters participating in this program were offered, by Bucher et al. [46], a reinforced healthy eating intervention program that included a one-hour educational workshop, one cooking class, and a one-hour individual coaching session with a dietician. Survey instruments (Dutch Eating Behavior Questionnaire and Intuitive Eating Survey) were used to assesses emotional eating, eating in response to environmental stimuli, restrained eating, eating for physical and not emotional reasons, permission to eat, and attention to satiety cues [46]. Weight and body composition were also used as a measure of analysis to evaluate the intervention. The diets of firefighters that participated in the study were unbalanced and associated with low intakes of fiber and micronutrients compared to national standards. Overall, the results indicated no significant changes in eating behavior or anthropometric data. Their results also suggested that the main barriers to developing healthier eating habits included lack of motivation, prioritization, and time [46]. It is important to note that the sample size of this study was small.

The First Twenty intervention (TF20) was developed by a SFF in 2008 with the goal of decreasing weight and improving health among SFFs and reducing the number of CVD-related on-duty deaths. This intervention program includes practical approaches to nutrition, fitness, and positive mental health; weekly messages delivered via email that highlights the TF20 program; and nutritional education focused on sufficient hydration, increasing consumption of fruit and vegetables, and reducing the intake of highly processed foods. In addition, a volunteer firefighter (VFF) trained in MI telephone-based health coaching addressed progress with the program, obstacles to improvement, and tailored ways of setting targets to change behavior. Day et al. [41] conducted a cluster randomized trial with crossover, utilizing the TF20 program. This study is unique because it was tailored specifically for volunteer SFFs, who not only have the same responsibilities as career SFFs but also often have a full-time or part-time primary job. Among all study participants, a weight reduction of 1.7 to 2.8 pounds was observed, and among overweight and obese participants a weight reduction of 2.3 to 3.1 pounds was observed at the end of the evaluation. The lack of program adherence among the treatment and control groups was a major limitation due to job constraints (e.g., SFF duties, missing attendance for physical assessments), as well as a lack of commitment to be fully engaged in the program. Departmental culture encouraging healthy eating and exercise was varied and may have influenced attrition. This highlights the importance of positive team culture towards healthy eating behaviors.

In addition to dietary training and education sessions, promotional pamphlets and messaging through social media apps have been used to encourage healthy eating habits of fruits and vegetables. Ng et al. [44] conducted an eight-week cluster randomized controlled trial in which they investigated the effects of using WhatsApp, a communication app, to promote healthy eating. The control group received a health promotion pamphlet, and the intervention group received the pamphlet as well as additional educational material through WhatsApp. The content of the material that Ng et al. [44] developed focused on the first four stages of the transtheoretical model (TTM) of behavioral change readiness (precontemplation, contemplation, preparation, and action) to explain the reasoning behind healthy eating habits, state the benefits of incorporating more fruits and vegetables into daily diets, provide various cooking methods for vegetables, and include practical advice for eating enough fruits and vegetables when dining out and eating during special occasions. These materials were sent to the intervention group in intervals of one to two weeks. The contents of the education pamphlet included material for the first four TTM stages, as well. At the conclusion of the study, participants from the intervention group reported high levels of satisfaction with the intervention since they were able to refer to the teaching materials at any time and in any place. For the intervention group, significant increase in the mean number of days that the participants consumed fruits and vegetables was observed. Only fruit consumption significantly increased among participants in the control group. Although the sample size was small, the results indicate that continuous reinforcement of knowledge about healthy eating behavior is important for achieving positive changes in diet. The study also demonstrates the feasibility of a practical approach to implement such intervention among SFFs.

The Mediterranean diet has become a very popular dietary pattern among individuals looking to become healthier and/or lose weight. Sotos-Prieto et al. [42] adapted the Mediterranean-based diet interventions and created a program called “Feeding America’s Bravest”. This program was tested in a cluster-randomized control trial that includes 44 fire stations and approximately 1000 SFFs within one city. Two groups were included in the intervention: (1) Mediterranean Diet Nutritional Intervention (MDNI); and (2) usual care (no intervention). In Phase I, conducted over a period of 12 months, one group received active monthly MDNI while the other group continued with their usual care. In Phase II, which covered the remaining 12 months, the two groups crossed over, where the first group continued a self-sustained care and the second group received six months of active MDNI plus six months of self-sustained continuation. The MDNI consisted of a study website, group educational sessions, and written materials to cultivate increased knowledge. This included a brochure with Mediterranean diet (MD) suggestions, recommendations for the food shopping list and sample recipes (including videos), and tips for practicing the MD at home and working with an MD pyramid unique to SFFs. This initiative involved a collaboration with a national chain grocery store in the U.S. (Kroger) to provide the participating SFFs and their families with discounted access to MD food. SFFs also had the option to sign up for 2–4 x/week emails or text messages that had positive reinforcement for healthy dietary and lifestyle principles. The purpose of these methods is to examine the sustainability of the Mediterranean diet principle. The intervention techniques were driven by Social Cognitive Theory, economic benefits, family and peer reinforcement, and environmental improvements. Study outcomes included changes in body composition, cardiometabolic risk markers, knowledge and self-efficacy, and variance in dietary changes. Although this style of diet intervention can be highly beneficial, efficacy results are still inconclusive. Program adherence and sustaining new habits are likely the leading factors that will help firefighters improve their health. Sotos-Prieto et al. [43] published a recent update on this cohort of SFFs regarding the effects of the MD on metabolic biomarkers. While only modest changes were observed, their results indicate that adherence to the MD resulted in favorable changes in biomarkers of lipid metabolism [43]. Although these results are promising, the small sample size limited the results of this study; therefore, future research performed with larger sample sizes is needed to confirm these results.

A more recent study by Almeida et al. [45] also used the MD for a six-week diet and exercise intervention to investigate the relationship between CVD risk reduction and diet adherence. The intervention was evaluated among a group of civilians and a group of municipal and volunteer firefighters. The MD intervention consisted of a training session specifying the types of foods to eat and portion sizes for each food group, as well as a diet manual specific to this study that included sample recipes, diet recording, food intake reporting, MD-relevant serving sizes, and health coaching [45]. An exercise protocol was also included in the intervention. The overarching hypothesis for this study was that firefighters, compared to civilians, would have worse cardiovascular health at the beginning of the study and poor diet adherence, but would have larger improvements in CVD risk markers after the intervention [45]. The results show that both groups had improved blood pressure and body composition; however, only the civilian group showed improvements in lipid levels and higher overall adherence to the diet. Although the firefighters did not have large improvements in CVD risk markers, as originally hypothesized by Almeida et al. [45], these results coupled with those from Sotos-Prieto et al. [42] and Sotos-Prieto et al. [43] suggest that adherence to the MD is associated with improved lipid levels and lower CVD risk and indicate that further research is needed to understand and evaluate the necessary factors to improve diet adherence among SFFs. 

## 4. Conclusions

The present narrative review provides an overview of the current literature on dietary behavior and interventions among SFFs. Dietary habits and patterns among SFFs still remain largely inconsistent and poor. The current literature suggests there is a need for improvement to create positive and sustainable changes in dietary behaviors among firefighters. Current diet practices are influenced by the strenuous and inconsistent nature of the work environment, creating an additional level of risk to the occupational exposure experienced by SFFs. The interactions between these factors are also unknown. Team-setting has a greater influence on successful interventions focused on improving firefighter diet, as indicated by results from the PHLAME studies. Therefore, future interventions should consider implementation across the entire firehouse or group of firefighters, rather than being at the individual level. Investigating details on how and why such interventions (e.g., education, diet, supplements) are effective would benefit future interventions and the improvement of current interventions. Background information is needed to understand key factors required to drive positive change, as well as develop solutions to unchangeable work variables (e.g., sleep disturbances, shift work). Longitudinal studies would be the most resourceful in attaining this information from firefighters. There are additional opportunities for improving dietary behavior outside of the SFFs working hours, which could make dietary changes during shift work more sustainable. To achieve this, future studies could use a two-phase intervention that includes an intervention at the firehouse level and an intervention with the SFFs’ families. Throughout this review, the literature on diet or nutritional education as part of training at the fire academies was found to be limited. However, it has been suggested by the existing research that intervention or educational programs beginning at the fire academy where SFF training begins could be impactful in establishing good dietary behavior across the entire firefighting career and positively changing the food culture among SFFs. Future observational studies examining diet trajectory across the firefighting career from recruitment to retirement could inform the development of an effective diet education for SFFs at fire academies. Results from such studies can inform solutions in reducing occupational risks among structural SFFs through healthier and modified dietary habits.

## Figures and Tables

**Table 1:** Observational Studies.

Author/Year	Study Design, Study Location	Study Objective	Study Population [Sample Size, Source Population, Age, Career Length, Sex, Race/Ethnicity]	Diet Pattern Assessment Tool	Key Results	Qualitative Narrative Assessment of Risk of Bias
Yang J et al., 2015 [17]	Cross-over; United States	To determine diet practice and diet preferences	*n* = 3127 (out of *n* = 3657); IAFF (professional) FF members; 42 ± 10 years; 92.6% male, 7.4% female; Race/ethnicity unspecified	19-question questionnaire about type of diet—18 diets listed	71% practiced no diet pattern, 9% Paleo, 8% low carbohydrate, 4% low fat, <2% commercial, 1% Mediterranean; 20.4% assessed self-knowledge of diet sufficient—dependent on and increasing with BMI; most (77.6%) disagreed with assertion that Fire Service provided sufficient diet education; Mediterranean diet is most preferred	Selection bias (self-selection, convenient sample); Information bias (reporting); Non-response bias; Non-differential misclassification
Eastlake A et al., 2015 [6]	Cross-sectional; Hamilton County, Ohio, United States	Determine baseline for prevention on cardiovascular disease and the relation to multiple risk factors	*n* = 157 (out of *n* = 1431); Hamilton County, OH FF members; average age 40.8 (range 19–72 years old); career length unspecified; 100% male; 90.5% White, 7.6% Black/African American, 0.6% Alaskan Native/American Indian, 1.3% Other	15-question questionnaire targeting occupational risk factors, lifestyle risk factors, and demographic characteristics.	83% of participants consumed red meat 1–5 times/week; 90% ate fast food 1–3 times/week; 57% ate fish <1/week; 73% ate vegetables 1–5/week; 92% ate grains 1–7/week; reduced risk for high cholesterol associated with increase alcohol consumption; consumption of whole grains is major variable in predicting reduced chance of CVD.	Selection bias (convenient sample); Information bias (reporting); Non-differential misclassification
Sotos-Prieto M et al., 2019 [18]	Cross-sectional; United States	Identify hindrances, challenges, and cost vs. time vs. social relation to food	Interviews: *n* = 12; source population all U.S.; mean age 49.2 ± 8.0 years; 19.4 ± 11.6 years career length; 91.7% male; 91.7% White, 8.3% Hispanic/Latino Focus Groups: *n* = 5; source population all U.S; mean age 50.8 ± 6.1 years; 19.4 ± 11.4 years career length; 100% male; 100% White	~30 min. interviews about current food environment, barriers, and solutions to improve diet and lifestyle habits; ~1 h focus group meetings to talk about themes from one-on-one interviews	Fire academies and firehouses had different food environments. Most interviewees commented positively to use of Mediterranean diet. Financial resources and knowledge, as well as no supportive culture from senior staff are barriers to improving nutrition. Nutrition education, incentives, and training to promote cohesive culture of health eating were supported by interviewees.	Selection bias (convenient sample); Information bias (reporting)
Haddock et al., 2015 [19]	Cross-sectional; United States	To present information on the patterns of alcohol consumption among firefighters.	*n* = 954 on patterns of p30 day alcohol use; *n* = 246 on off-duty alcohol intake from 24 hr recall; FF departments across U.S. region; 39.1 ± 8.8 years old; 14.2 ± 8.6 years career, 100% male; 76.4% White	Questionnaire modelled after common substance use questions on surveys such as the national Household Survey on Drug Abuse and on surveys of military members.	Rank was related to OR of excessive drinking and episodic heavy drinking (FF > chief); Excessive and sporadic heavy drinking are commonly related to years of service (fewest > most); years of service inversely related to # of drinks and prevalence of episodic heavy drinking on selected off-duty day.	Selection bias (voluntary participation); Information bias (recall); Differential misclassification; Non-differential misclassification
Yang et al., 2014 [20]	Cross-sectional; United States	Investigate effects of adherence to the Mediterranean diet and relations to CVD biomarkers, metabolic syndrome, and physical body characteristics.	*n* = 780; two midwestern U.S. states; 35.6 ± 10 y.o. normal weight, 372.2 ± 8.4 y.o. overweight, 38.9 ± 8 y.o. obese class I, 38.6 ± 8.1 y.o. obese class II/III; career length unspecified, 100% male; Race/ethnicity unspecified	Lifestyle questionnaire with a modified Mediterranean diet score (mMDS) on a scale of 0 (no conformity) to 42 (maximal conformity)	Obese subjects had lower mMDS; greater mMDS inversely related to risk of weight gain over past five years and presence of metabolic syndrom components; higher HDL-c and lower LDL-c in participants correlated with higher mMDS.	Selection bias (convenient sample); Information bias (recall, reporting); Non-differential misclassification
Anderson et al., 2017 [21]	Cross-sectional; United States	To determine firefighters’ perception of cancer risk	*n* = 100 (observation over 150 hrs), *n* = 17 (focus group); age in observation unspecified; focus groups age 29–58 y.o. *w*/average age of 51; ≥3 years career; Gender unspecified; Race/ethnicity unspecified	Interview with comment and informal questions; observations for attitude and behavior, environment, barriers, and chances for change; emerging themes used to discuss in focus groups	How healthy a meal is determined by the influence of who is cooking; constraints of occupation can play a role in diet and level of healthiness.	Selection bias (convenient sample); Information bias (reporting); Observer bias
Chatzitheodoridis et al., 2017 [22]	Cross-sectional; Greece	To examine BMI and obesity rates among Greek firefighters and observe nutrition habits and risk factors related to obesity.	*n* = 190 (*n* = 59 normal bmi, *n* = 95 overweight bmi, *n* = 36 obese bmi); Central and Western Macedonia; 37.83 ± 6.018 y.o. normal, 39.51 ± 7.419 y.o. overweight, 43.03 ± 6.056 y.o. obese; Career length unspecified; 94.2% male; Race/ethnicity unspecified	28 questions regarding diet, exercise, and work. Questions on a 5 or 7-point Likert type scale.	Consensus that there is need for exercise/nutrition program for SFFs. Economic crisis in country has affected how grocery purchase decisions are made.	Selection bias (voluntary participation); Information bias (reporting); Non-differential misclassification
Robertson et al., 2017 [23]	Cross-sectional; Canada	To assess the physiological demands and dietary habits of Canadian firefighters during operations.	*n* = 21 (out of *n* = 72 potential); northern Ontario Fire Base; 29.8 ± 8.5 y.o.; career length unspecified; 100% male; Race/ethnicity unspecified	Food logs	Sufficient kilocalories for deployment not met; SFFs exceed fat intake and failed to meet recommended carbohydrate intake. Protein intake acceptable.	Selection bias (convenient sample); Information bias (reporting)
Friel et al., 1988 [24]	Cross-sectional; United States	To observe if diets of FF would predispose them to risk of heart disease.	*n* = 35; St. John’s Fire Department; Age, career length, sex, and race/ethnicity unspecified	Food log and dietary data base	FFs on shift appear to consume more fat, cholesterol, protein, sodium, and potassium than is recommended or than they do at home; average FF in sample was classified as overweight or obese.	Selection bias (convenient sample); Information bias (reporting)
Romanidou et al., 2020 [25]	Cross-sectional; United States	To examine if and what associations exist between a modified Mediterranean Diet Score and anthropometric indices, blood pressure, and biochemical parameters	*n* = 460; 44 Indianapolis fire stations and 6 Fishers, Indiana fire stations enrolled in Feeding America’s Bravest; 46.7 ± 8.3 y.o.; career length unspecified; 94.4% male; 85.5% White, 12.5% African American, 1.9% Other	131-item semiquantitative food frequency questionnaire and modified Mediterranean Diet Score (a validated instrument for measuring adherence to the Mediterranean diet)	Increased adherence to the Mediterranean diet was associated with markers of lower cardiometabolic risk.	Selection bias (convenient sample); Information bias (reporting); Non-response bias; Non-differential misclassification
Johnson & Mayer 2020 [26]	Cross-sectional; United States	To determine whether firefighters are meeting recommended guidelines of the Military Dietary Reference Intakes (MDRI)	*n* = 150; 13 career fire departments in Southern California enrolled in the Regional Firefighter Wellness Initiative; 37.35 ± 8.44 y.o.; career length unspecified; 100% male; Race/ethnicity unspecified	Three-day food record and lifestyle questionnaire	Compared to MDRI reference values, firefighters consumed an inadequate amount of total calories, linolenic and alpha-linolenic fatty acid, fiber, vitamins D, E, and K, potassium, magnesium, zinc, and carbohydrates. Vitamin D, magnesium, and potassium had the greatest shortcomings	Selection bias (convenient sample, voluntary participation); Information bias (recall, reporting); Differential misclassification
Vatandoost et al., 2020 [27]	Cross-sectional; Iran	To investigate the association between dietary inflammatory index (DII) and risk of cardiovascular diseases (CVD) among firefighters in Tehran	*n* = 273; Tehran fire stations; 33.99 ± 6.34 y.o.; career length unspecified; 100% male; Race/ethnicity unspecified	168-item semiquantitative questionnaire	Participants with higher DII scores tend to ingest more fat, saturated fat, and less PUFA, MUFA, EPA, and DHA. Several vitamins and minerals (including A, E, K, B1, B2, B3, B6, B9, C, magnesium, zinc, and calcium) were also found to be decreased in those with higher DII scores.	Information bias (reporting); Non-differential misclassification
Hershey et al., 2020 [28]	Cross-sectional; United States	To determine if there is an association between anthocyanin intake and physical activity on lipid profile measures	*n* = 249; Feeding America’s Bravest trial; 47.2 ± 7.4 y.o.; career length unspecified; 95% male; Race/ethnicity unspecified	131-item semiquantitative food frequency questionnaire	Anthocyanins were inversely associated with total cholesterol:high-density lipoprotein (HDL) cholesterol.	Selection bias (convenient sample); Information bias (reporting); Non-differential misclassification
Bonnell et al., 2017 [29]	Fixed mixed method; Australia	To explore factors influencing food choice and dietary intake in shift workers	Focus group *n* = 41, Dietary recall *n* = 19; Melbourne fire stations; 36 y.o.; career length unspecified; Focus group 97.6% male, Dietary recall 94.7% male; Race/ethnicity unspecified	Focus groups were conducted using semi-structured open questions and 24 hr dietary recalls via telephone interviews	Unhealthy dietary behaviors were identified among shift workers, specifically night shift that include an increase of discretionary foods and lack of availability of healthy food choices at night	Selection bias (convenient sample, voluntary participation); Information bias (recall, reporting); Differential misclassification
Gupta et al., 2020 [26]	Case-control; Australia	To investigate the impact of heat and sleep restriction on snacking behavior and food cravings	*n* = 66; Southern Australian fire stations; Control 39 ± 16 y.o., Sleep Restricted 39 ± 15 y.o., Hot 36 ± 13 y.o., Hot & Sleep Restricted 41 ± 17 y.o.; Career length unspecified; 84.8% male; Race/ethnicity unspecified	Food records and hunger/cravings questionnaire	Sleep restriction and heat did not impact feelings of hunger and fullness across the day and did not lead to greater cravings for snacks. These findings suggest that under various simulated firefighting conditions, it is not the amount of food that differs but the timing of food intake.	Selection bias (controls selection); Information (reporting); Non-differential misclassification
Hershey et al., 2021 [30]	Cross-sectional; United States	To assess the association between the Mediterranean lifestyle and metabolic syndrome	*n* = 249; Feeding America’s Bravest trial; Age (1st Tertile 46.92 ± 6.98 y.o., 2nd Tertile 46.66 ± 7.57 y.o., 3rd Tertile 46.56 ± 8.08 y.o.); Career length unspecified; Sex (1st Tertile 97.8% male, 2nd Tertile 92.9% male, 3rd Tertile 93.3% male); Race/ethnicity unspecified	131- item semi-quantitative 2007 grid Harvard food-frequency questionnaire (FFQ), also known as the Willett FFQ	Higher adherence to traditional Mediterranean lifestyle habits, as measured by a comprehensive MEDLIFE index, was associated with a lower prevalence of metabolic syndrome and a more favorable cardiometabolic profile.	Selection bias (convenient sample); Information bias (recall, reporting); Non-differential misclassification
Kadiwar et al., 2021 [31]	Cross-sectional; United States	To characterize the diet of volunteer firefighters compared with the United States recommended dietary intake	*n* = 122; New Jersey Firefighter Cancer Assessment and Prevention Study (CAPS); Age (17.9% < 30 y.o., 29.5% 30–39.9 y.o., 24.1% 40–49.9 y.o., 28.6% ≥ 50 y.o.); Career length (25% < 10 yeas., 25.9% 10–≤25 yeas, 26.8% 25–≤40 yeas., 22.3% ≥ 40 yeas.); 100% male; 96.4% non-Hispanic white	Dietary Screener Questionnaire	Participants had lower mean intakes of fruit and vegetables, whole grains, and dietary fiber, and a higher mean intake of added sugars compared with the U.S. recommended dietary intake.	Selection bias (convenient sample); Information bias (reporting); Non-differential misclassification

**Table 2:** Intervention Studies.

Author/Year	Study Design, Study Location	Study Objective	Study Population [Sample Size, Age, Career Length, Sex, Race/Ethnicity]	Type and Length of Intervention	Outcome (If Any)	Key Results	Qualitative Narrative Assessment of Risk of Bias
Waldman HS et al., 2019 [32]	Cross-over; United States	Effect of 28-day carbohydrate restricted diet on performance and cardiometabolic markers	*n* = 15 (from 21 initial persons); 33.5 ± 9.7 years; 7.9 ± 7.4 years; 100% male; Race/ethnicity unspecified	Carbohydrate restricted; 28 days	Cardiometabolic and performance parameters	Average body weight reduced by 2.7 kg; respiratory exchange rate reduced; SBP and DBP reduced; rating of perceived exertion reduced	Selection bias (convenient sample, small sample); Information bias (reporting); Carryover effects; Differential misclassification
Sotos-Prieto et al., 2017 [7]	Cluster-randomized intervention; United States	Change fire departments’ food culture; Compare Mediterranean Diet Nutritional Intervention (MDNI) (group 1) vs. usual care (group 2; control) and its outcomes.	*n* = 1000 (from 44 fire stations), 18 years old or older, all career firefighters (no length listed), 95% Male; Race/ethnicity unspecified	Applied the Mediterranean diet at work and at home for 12 months; In Phase II of the remaining 12 months, group 1 no longer received active MDNI and continued a self-sustained continuation, while group 2 crossed over to receive six months of active MDNI and six months of self-sustained continuation.	None	Increase knowledge; increase self-efficacy; normalized healthy behaviors; navigate barriers to discounted food access	Selection bias (convenient sample); Information bias (recall, reporting); Carryover effects; Noncompliance/Loss to follow-up
Ranby et al., 2011 [33]	Balanced randomized trial; United States	Examine the process where PHLAME improved healthy eating and physical activity among firefighters	*n* = 397 (from 48 stations), mean age of 41 years old, no career length provided, 93% Male; 91% White	Health Promotion Intervention, 12-month intervention with 1 year follow-up	Increase knowledge and self-efficacy about fruit/vegetable consumption and exercise compared to controlled PPTs.	Team participants post-intervention had increased fruit/vegetable consumption; increased knowledge of health benefits from fruit/vegetable consumption; improved dietary environment among coworkers.	Selection bias (convenient sample, control selection, non-independent samples); Information bias (recall, reporting); Loss to follow-up; Exposure misclassification
Elliot et al., 2007 [34]	Randomized trial; United States	Assess and compare PHLAME’s (Promoting Healthy Lifestyles: Alternative Models’ Effects) two health promotion methods	*n* = 599; 41 ± 9 years; 16± 9 years; 579 men (97%) and 20 women; 91% White	The PHLAME study compared (1) a team-based curriculum, (2) motivational interviewing that is one-on-one. Used social cognitive theory components as well; 12 months	Fruit and vegetable consumption, daily physical activity, and adequate body weights.	Increased fruit and vegetable consumption among team and MI; increased general well-being; significantly less weight gain.	Selection bias (convenient sample, non-independent samples); Information bias (recall, reporting); Loss to follow-up; Exposure misclassification
Gill et al., 2019 [35]	Cluster-randomized controlled clinical trial; United States	Test the feasibility of a lifestyle intervention aimed to improve risks associated with CVD.	*n*= 96 for treatment, *n* = 79 for control; treatment 43.02 ± 8.25 years and control 41.77 ± 9.68 years; no career length listed; treatment 89.58% male and control 97.46% male; Race/ethnicity unspecified	Personalized diet and exercise plan; 12 months	HDL level measurements; physical and biological characteristics of the body, e.g., weight, body fat, circumference of waist/hip, glycemic level, insulin resistance.	Significant reduction of body weight and waist circumference; Increased a1 HDL; Decreased triglyceride and insulin concentrations; Program adherence with weight loss and increases in ⍺1HDL	Selection bias (control selection); Information bias (recall, reporting)
Macedo et al., 2015 [36]	Randomized trial (controlled double-blinded); Brazil	Determine the plasma metabolic response and oxidative stress indicators in Brazilian military firefighters	*n* = 60; Placebo 22.3 ± 1.78 years treatment 21.46 ± 1.77 years; no career length listed; 100% male; Race/ethnicity unspecified	Resveratrol (RES) supplementation; 90 days	RES supplements did not induce any changes regarding hepatic consequences.	IL-6 and TNF-a levels reduced after three months of RES supplements. Physical fitness test did not challenge antioxidant defense system, so no tangible results.	Selection bias (convenient sample); Exposure misclassification
McDonough SL et al., 2015 [37]	Cohort intervention; United States	(1) To determine the feasibility of an 8-week wellness program; (2) To test the effect of “FIT Firefighter” intervention on cardiovascular fitness.	*n*= 29; 38.6 ± 5.5 years; no career length (just said they were all fulltime); Sex and race/ethnicity unspecified	Nutrition education, health coaching, and strength and fitness condition; 8-weeks	Changes in physical characteristics of the body, i.e., BP, resting HR, aerobic fitness, circumference of waist, body weight.	Improved behavior changes regarding health and nutrition. Improvement in physical characteristics.	Selection bias (convenient sample); Information bias (reporting); Exposure misclassification
Pirlott et al., 2012 [38]	Randomized trial; United States	Report findings to understand the nuances of how an MI counselor speaks during the PHLAME intervention.	*n* = 202; 41 ± 7.46 years; no career length listed; 98% male; 95% White	Randomized prospective worksite trial; 12 months	Daily fruit and vegetable consumption.	Increased consumption of fruits and vegetables. MI Counselor empathy and consistent behavior indicates better influence on SFF’s increased consumption of fruits and vegetables	Selection bias (convenient sample, non-independent samples); Information bias (recall, reporting); Exposure misclassification
Goheer A. et al., 2014 [39]	Cohort Study; United States	Create, introduce, and evaluate nutrition intervention to reduce adverse health risks among firefighters.	*n* = 90; Age, career length, sex, and race/ethnicity unspecified	Intervention contained education sessions (written and visual) to demonstrate healthier nutrition; 6 months with a 1-year follow up	No outcomes listed	Majority report improved changes in food environment at firehouse and personal home. Education sessions and demonstrations were highest rated for most helpful in the intervention.	Information bias (reporting); Noncompliance
MacKinnon DP et al., 2010 [40]	Randomized trial; United States	Describe immediate and sustainable effects of health intervention programs for structural firefighters.	*n* = 599; MI = 41 ± 8.9 years, TEAM = 39.3 ±8.7 years, and control = 41.3 ± 8.8 years; MI = 16.4 ± 8.5 years, TEAM 14.4 ± 8.7, and control 15.7 ± 8.9; 579 men (97%) and 20 women; 91% White	(a) A team-based, peer-led scripted health promotion curriculum, (b) one-on-one motivational interviewing health coaching, and (c) a testing-and-results-only comparison condition; baseline, 2 intervention years, and 4 follow-up years	Intervention behavioral objectives: (1) minimum consumption of 5 servings of fruits and vegetables, (2) being physically active for minimum 30 min a day, (3) maintaining a healthy body weight	High willingness to try new food and preparation at the firehouse and personal home. Team-based curriculum more effective on nutritional behavior and physical fitness at one year follow-up compared to one-on-one MI.	Selection bias (convenient sample, non-independent samples); Information bias (reporting); Loss to follow-up; Exposure misclassification
Day et al., 2019 [41]	Randomized control trial with crossover; United States	Evaluate efficacy of tailored weight loss intervention among volunteer firefighters.	Treatment *n* = 217; Control *n* = 178; VFF all over U.S.; treatment age 37.3 ± 12.7, control age 36.9 ± 12.6; career length for treatment 10.5 ± 9.9, control 11.0 ± 10.0; treatment 77.5% male, control 83.6% male; Race/ethnicity unspecified	A web-based health and wellness program called “The First Twenty”; contains online nutrition education, recommendations for physical activity, and encourages mental health applications; 12 months	Weight loss	Participants experienced 1.7–2.8 pounds of weight loss after the intervention concluded. There is still a need to improve adherence to program.	Selection bias (control selection); Information bias (reporting); Carryover effects; Exposure misclassification
Sotos-Prieto M et al., 2019 [42]	Cohort (nested); United States	To determine the validity of mMDS vs. biomarkers and FFQ for determining Mediterranean diet practice	*n* = 48; 24 on self-sustained Mediterranean diet after 12-month education intervention; 24 on active education intervention after 12 months of no intervention; Indianapolis FD; 47.52 ± 7.63 years (48.36 ± 8.29 years in parent study); 92.7% male; 82.8% White, 7.2% African American, 3.2% Other	13-domain Modified Mediterranean Diet Scale (mMDS), FFQ-derived mMDS, FFQ after 12 months	Assessing the concordance between questionnaires and adherence to Mediterranean Diet.	mMDS items (olive oil intake, choice of olive oil as type of fat, overall mMDS score) correlated with omega-3 PUFA; FFQ nutrient intake correlated with DHA and EPA at baseline and at follow-up (r = 0.624–0.775)	Selection bias (convenient sample, non-independent sample); Information bias (recall, reporting); Noncompliance/Loss to follow-up; Exposure misclassification
Sotos-Prieto et al., 2020 [43]	Cluster-randomized controlled trial (nested); United States	To investigate the longitudinal effects of the Mediterranean diet on metabolic biomarkers	Group 1 Int. *n* = 24, Group 1 Cont. *n* = 24, Group 2 Int. *n* = 22, Group 2 Cont. *n* = 22; Feeding America’s Bravest trial; Group 1 Int. 47.5 ± 6.7 y.o., Group 1 Cont. 47.6 ± 8.6 y.o., Group 2 Int. 45.9 ± 6.7 y.o., Group 2 Cont. 49.9 ± 8.4 y.o.; Career length not specified; Group 1 Int. 91.7% male, Group 1 Cont. 95.8% male, Group 2 Int. 84.6% male, Group 2 Cont. 94.1% male; Race/ethnicity unspecified	Mediterranean Diet Intervention (MedDiet intervention) consisting of educational sessions and videos, leaflet and recommendations, firefighter-tailored Mediterranean, in-site chef cooking demonstration, a firefighters’ food pyramid, Mediterranean food samples and discount coupons to a large supermarket chain for specific Mediterranean Diet-compatible foods. 12 months	Analysis of plasma metabolic biomarkers, assessment of adherence to Mediterranean diet via the Mediterranean Diet Score and PREDIMED score	The MedDiet intervention led to favorable changes in biomarkers related to lipid metabolism, including lower LDL-C, ApoB/ApoA1 ratio, remnant cholesterol, M-VLDL-CE; and higher HDL-C, and better lipoprotein composition.	Selection bias (convenient sample, non-independent sample); Non-differential misclassification; Differential misclassification; Noncompliance/Loss to follow-up
Ng et al., 2021 [44]	Cluster randomized controlled trial; Hong Kong	To explore the feasibility of a promotion pamphlet and/or WhatsApp as a suitable mode of delivery to promote healthy eating habits with fruit and vegetables	Int. group *n* = 20, control group *n* = 25); 23 fire stations in Hong Kong; Age 35.0 ± 9.6 y.o. (int. group 32.9 ± 9.5 y.o., control group 36.6 ± 9.6 y.o.); Career Experience 11.3 ± 9.9 yeas (Intervention 9.4 ± 9.6 yeas, Control 12.8 ± 10.1 yeas); 100% male; Race/ethnicity unspecified	Health promotion pamphlet and teaching materials through WhatsApp; 8 weeks	Assessment of eating habits, specifically fruits and vegetables, as well as retention, practicality, and implementation of the intervention	There were significant improvements in the mean numbers of days consuming fruits and vegetables in the intervention group, and for fruit consumption in the control group.	Selection bias (convenient sample); Information bias (recall, reporting); Noncompliance/Loss to follow-up
Almeida et al., 2022 [45]	Two-group (Firefighters v. Civilians) diet and exercise pre-post intervention; United States	To examine the relationship between diet adherence and cardiovascular disease risk-reduction between civilians and firefighters with a 6-week Mediterranean diet and tactical training intervention	Firefighters *n* = 40, Civilians *n* = 30; Age (Civilians 41.6 ± 15.8 y.o.; Firefighters 39.6 ± 14.7 y.o.); Career length mean 18.8 years; Sex (Civilians 36.7% male, Firefighters 92.5% male); Race/ethnicity unspecified	Training session explaining the modified Mediterranean diet, diet manual, sample tracking sheets, sample recipes, and information on types of foods, access to a study website, and a ‘health coach’. 6 weeks	No outcomes specified	Both groups had improvements in blood pressure and body composition. However, the civilian group overall had better adherence to the diet.	Selection bias (convenient sample); Information bias (reporting)
Bucher et al., 2019 [46]	Pre- and post-intervention design; Switzerland	To assess the eating habits of firefighters, compare them with national guidelines, and evaluate the impact of this prevention program	*n* = 28; Swiss airport firefighters; Age 40.2 ± 6.3 y.o.; Career length 17.0 ± 6.3 y.o.; 100% male; Race/ethnicity unspecified	1 h educational workshop and a cooking class, as well as 1 h of individual coaching from a dietitian; 1 year	Assessment of anthropometric measurements	Intervention did not impact eating habits or anthropometrics at the group level. Main reported barriers for healthy eating were lack of motivation, prioritization, or time.	Selection bias (convenient sample, small sample); Information bias (reporting); Differential misclassification
Waldman et al., 2020 [47]	Cross-over; United States	To examine the effects of a 28-day, nonketogenic, carbohydrate-restricted diet on markers of inflammation, oxidative stress, and heart disease, using the AHA guidelines for risk stratification in professional firefighters	*n* = 15; Mean age not specified but age range was 20–45 y.o.; Career length not specified; 100% male; Race/ethnicity unspecified	Intervention was a nonketogenic, carbohydrate-restricted diet. 28 days	Anthropometric measurements; blood analysis; assessment of macronutrient breakdowns	Compared with baseline, the carbohydrate-restricted diet resulted in dramatic improvements to subjects’ cardiometabolic profiles.	Selection bias (convenient sample, small sample); Information bias (reporting); Carryover effects; Differential misclassification

## Data Availability

Not applicable.
